# Clinically silent LINE 1 insertion in the *PNPLA3* gene may impede genotyping of the p.I148M variant

**DOI:** 10.1038/s41598-021-00425-0

**Published:** 2021-10-22

**Authors:** Martin Leníček, Václav Šmíd, Petr Pajer, Anna Nazarova, Karel Dvořák, Iva Subhanová, Radan Brůha, Libor Vítek

**Affiliations:** 1 Institute of Medical Biochemistry and Laboratory Diagnostics, First Faculty of Medicine and General University Hospital in Prague, Charles University, Kateřinská 32, 12108 Prague, Czech Republic; 2 4th Department of Internal Medicine, First Faculty of Medicine and General University Hospital in Prague, Charles University, U Nemocnice 499/2, 12808 Prague, Czech Republic; 3 Department of Microbiology and Biological Defence Research, Military Health Institute Prague, Tychonova 1, 16001 Prague, Czech Republic; 4 Department of Gastroenterology and Hepatology, Regional Hospital Liberec, Husova 357/10, 46063 Liberec, Czech Republic

**Keywords:** Biochemistry, Genetics, Molecular biology, Gastroenterology

## Abstract

The patatin-like phospholipase domain containing 3 (*PNPLA3*) gene (viz. its I148M variant) is one of the key players in the pathogenesis of nonalcoholic fatty liver disease (NAFLD). We have identified a novel insertion/deletion variant of 1114 bp, localized in the second intron of the *PNPLA3* gene, which corresponds to the 3′ terminal sequence of the long-interspersed element (LINE-1). DNA analysis of 122 NAFLD patients and 167 control subjects as well as RNA analysis of 19 liver biopsies revealed that the novel variant is very common (frequency = 0.41), fully linked to the clinically important I148M variant, and clinically silent. Although the LINE-1 insertion does not seem to have any biological effect, it can impede genotyping of the I148M variant. If insertion prevents the attachment of the diagnostic primer, then the non-insertion allele will be selectively amplified; and thus the frequency of the 148M "risk" allele will be significantly overestimated due to the complete linkage of the LINE-1 insertion and the 148I allele of the *PNPLA3* gene. Therefore, our findings underline the importance of careful design and consistent documentation of the methodology, including primer sequences. Critical revisions of the results of some studies that have already been reported may therefore be needed.

## Introduction

Nonalcoholic fatty liver disease (NAFLD), the liver manifestation of metabolic syndrome has become the leading chronic liver disease worldwide, and is going to represent a major health burden in the near future^1^. Although the incidence of NAFLD closely correlates with both the Western diet and lifestyle that are associated with being overweight and obesity, inter-ethnic differences together with the existence of “lean NAFLD” patients underlines the effect of a genetic background.

In 2008, a large genome-wide association study showed that the common variant (I148M, rs738409) in the patatin-like phospholipase domain containing 3 (*PNPLA3*) gene was strongly associated with a higher hepatic fat content and contributed to a susceptibility to NAFLD^2^. Additionally, the 148M allele has also been demonstrated to represent an important risk factor for development of alcohol-related cirrhosis^3^. The observation that I148M substitution markedly reduced the triacylglycerol hydrolase activity of PNPLA3 seemed to clearly explain the pathophysiology of liver fat accumulation^4^. The disrupted fat hydrolysis theory was challenged by the observation that *pnpla3* KO mice do not present with a NAFLD-like phenotype^5,6^. The study by Smagris et al*.*^7^ showed that the *pnpla3* 148M allele knock-in mice develop hepatic steatosis; suggesting that not only loss-of-function, but that gain-of-function also plays a role. It was later shown that the 148M variant of PNPLA3 escapes proteasomal degradation and accumulates on hepatic lipid droplets^8^. There it sequesters α-β hydrolase domain containing 5 (ABHD5, CGI-58), which is essential for the proper action of adipose triglyceride lipase (ATGL, PNPLA2)^9^. Such impaired lipase activity appears to at least partially explain PNPLA3-triggered steatosis^9,10^. Finally, the recent study on human hepatocytes differentiated from pluripotent stem cells favors loss-of-function theory with increased susceptibility to xenobiotics^11^. Although the precise mechanism of PNPLA3 related susceptibility to NAFLD remains uncovered, the I148M variant is back as a key genetic determinant of hepatic fat content, which should not be overlooked in any NAFLD related study.

In our cohort of NAFLD patients we detected several individuals that were resistant to polymerase chain reaction (PCR) amplification of *PNPLA3*. PCR failure in those patients was specific to the *PNPLA3* region alone, and could not be explained by degraded or poor quality DNA; suggesting that a novel DNA alteration may be involved. Therefore, the aim of this study was to identify the cause of PCR failure and to assess its possible clinical function.

## Results

### Detection of the novel variant

**Figure 1:**
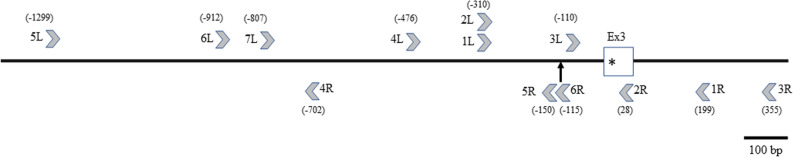
Primer location. Grey arrows show the relative position of forward (up) and reverse (down) primers used to localize the Insertion in the PNPLA3 gene. Numbers in parentheses show the position of primers relative to the p.I148M variant. White box indicates the third exon; asterisk indicates the location of p.I148M variant (position 5043 in NG_008631.1, corresponding to 43,928,847 in GRCh38.p13 assembly, accession NC_000022.11); black vertical arrow indicates the location of the Insertion (position 4921/4922 and 43,928,725/6 in NG_008631.1 and NC_000022.11, respectively).

When setting up the PCR-restriction fragment length polymorphism (RFLP) method for detection of the I148M variant in the third exon of the *PNPLA3* gene (using primers 1L and 1R, sequences are listed in Supplementary Table 1), repeatedly the PCR did not show any amplification in two patients (further referred as probands). As neither spectrophotometry nor electrophoresis suggested poor DNA quality, and several other sequences were successfully amplified using the probands’ DNA as a template, we suspected at least one primer annealed to a polymorphic site. Therefore, we tested PCR amplification using combinations of three additional left (2L, 3L, and 4L) and two right (2R, 3R) primers (see Fig. 1 for primer locations). While all tested primer combinations yielded specific amplicons in the control samples, in the probands they were only obtained when the forward primer 3L was present. On the other hand, the choice of the reverse primer did not play any role. This suggested that the cause of the PCR failure lies at least 110 bases upstream from the I148M variant. To localize this more precisely, we attempted PCR amplification of the suspected area using a combination of the other 6 primers (three left—5L, 6L, 7L; and three right—4R, 5R, 6R). In both probands, all reactions containing the reverse primer 6R were negative, while the other combinations produced the specific amplicon. This strongly suggested a larger insertion about 110–150 bp upstream of the I148M variant. We amplified this region using Long Range PCR (primers 1L + 2R; 4L + 2R) in the probands and control subjects. In both probands, the size of the PCR product was more than 1 kb longer than expected (than that observed in the controls); finally proving the presence of a substantial insertion (further referenced as Insertion or Ins allele).

### Characterization of the insertion

**Figure 2:**
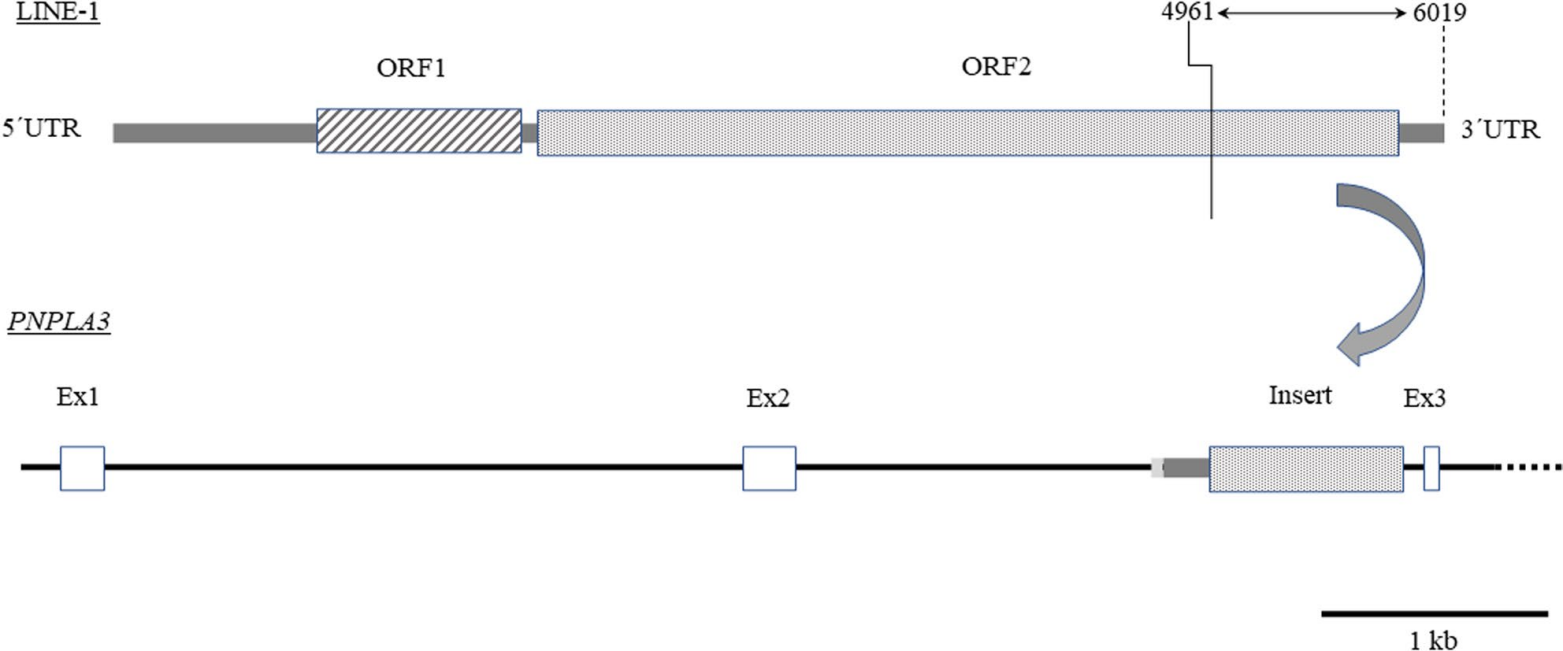
Structure of Insertion. The typical LINE-1 element consists of two open reading frames (ORF, hatched and dotted boxes) and 5′ and 3′ untranslated regions (UTR, thick gray line). The extent (horizontal double arrow, numbers indicate position in the “LINE-1 reference” AF148856.1), position, and orientation of the LINE-1 insert in the second intron of PNPLA3 is shown. 5′UTR and introns of PNPLA3 are shown as a black line (the dotted line represents 3′portion of PNPLA3, which is not shown), and exons as white boxes. The Insert consists of a short filler sequence, 3′UTR (thick light and dark gray lines, respectively), and part of ORF 2 (dotted box) of the LINE-1 element.

PCR products from both probands were sequenced using Sanger sequencing (using the same primers). As part of the sequence was illegible (likely to one or more homopolymeric regions), the PCR product was also re-sequenced by the Oxford nanopore sequencing method. We found that a short sequence (tgcaaagggcattttc) located about 100 bp upstream from exon 3 was duplicated, and that an insert of 1114 bp was placed between the duplications. After a short difficult-to-sequence "filler" region ((t)_14_ggtgatg(t)_34_—sequence may not be entirely accurate; namely the exact length of the homopolymeric regions), the remaining sequence showed a perfect match with the 3′ terminal sequence of the LINE-1 element in antisense orientation (Fig. 2).

### Insertion frequency, haplotype and association with NAFLD

The fact that both unrelated probands were homozygous for the Insertion suggests that this variant may not be rare. In fact, genotyping of 167 unrelated healthy Caucasian controls showed that the Ins allele was surprisingly common, with a frequency of 0.41. Given the high frequency of the Insertion, we wondered whether it can have any impact on *PNPLA3* expression/function, and whether it plays any role in the pathophysiology of NAFLD. Case–control comparison showed that unlike the 148M allele, the Insertion was not associated with NAFLD (Table 1). No clear contribution of the Insertion was observed when haplotypes were constructed for both variants; as all significant differences were attributable to the I148M variant (Table 2).

**Table 1:** Association of I148M and WT/Ins with NAFLD. Unlike I148M, the WT/Ins variant is not associated with NAFLD either in the allele or in the genotype analysis. Number of subjects/chromosomes are shown, frequencies are in brackets. Results are expressed as OR [5–95%].

I148M	Allele		Genotype		
	I	M	I/I	I/M	M/M
NAFLD	156 (0.639)	88 (0.361)	50 (0.41)	56 (0.459)	16 (0.131)
Control	249 (0.746)	85 (0.254)	94 (0.563)	61 (0.365)	12 (0.072)
*OR = 0.61 [0.42–0.87], p = 0.006*			p = 0.025		

WT/Ins	WT	Ins	WT/WT	WT/Ins	Ins/Ins
NAFLD	150 (0.615)	94 (0.385)	45 (0.369)	60 (0.492)	17 (0.139)
Control	196 (0.587)	138 (0.413)	54 (0.323)	88 (0.527)	25 (0.15)
*OR = 1.12 [0.80–1.57], p = 0.499*			p = 0.723

**Table 2:** Association of WT/Ins-I148M haplotype with NAFLD. WT-148I haplotype is protective, while WT-148M increases the risk of NAFLD. Both effects are attributable to the I148M variant itself, with no contribution of the WT/Ins variant. Number of subjects (haplotype frequencies) are shown. Results are expressed as OR [5–95%].

	WT-148I	WT-148M	Ins-148I	Ins-148M
NAFLD	62 (0.254)	88 (0.361)	94 (0.385)	0
Control	111 (0.332)	85 (0.254)	138 (0.413)	0
OR [95% CI]	0.68 [0.47–0.99]	1.65 [1.15–2.37]	0.89 [0.64–1.25]	na
	p = 0.043	p = 0.006	p = 0.499	na
	Global p = 0.026			

Interestingly, not a single haplotype consisting of the Insertion and 148M alleles was observed. This, together with the very short distance of both variants, suggested strong linkage disequilibrium. This was confirmed both in silico by haplotype analysis (D′ = 1, r2 = 0.286) in 289 unrelated individuals (cases and controls) as well as in vitro by allele specific PCR–RFLP (done both for the Insertion and wild type (WT) allele) in 71 compound heterozygotes. In all cases, the Insertion was located on DNA carrying the I (WT) allele of I148M (although the I148 allele not carrying the Insertion exists). For a complete overview of genotypes see Supplementary Table 2.

### Transcript analysis, quantitative RT PCR

The possible Insertion-mediated transcript alteration (alternative splicing, exon skipping, etc.) was evaluated by long-range PCR (spanning from exon 2 to exon 8, primers Ex2L and Ex8R) in liver cDNA samples in two homozygous carriers of the Ins allele and two WT controls. Only single PCR products, of the expected size, were obtained in all subjects. Overall liver PNPLA3 expression (relative to HPRT; primers Ex2L vs. 3L, and HPRT L vs. HPRT R) was not significantly different in subjects carrying at least one Ins allele (n = 12) when compared to WT homozygotes (n = 7): 145% (115–227%) vs. 100% (60–219%), p = 0.3. The possible premature termination of *PNPLA3* transcription was assessed by quantification of the 3′ ends (exon 7/8 boundary, primers Ex7L and Ex8R) of the transcript (relative to its 5′ end, exon 1/2 boundary, primers Ex1L and Ex2R). The relative abundance of 3′ends in Ins allele carriers (n = 12) was not different from those in the WT homozygotes (n = 7): 93% (74–98%) vs. 100% (61–134%), p = 0.43. To further test the possible effect of the Insertion on transcript stability, we performed allele specific qPCR (primers Ex2/3L-I(M) vs. Ex4R) in I148M heterozygotes with (n = 3) or without (n = 4) the Insertion. The abundance of the 148I allele (relative to the 148M allele) in subjects with/without the Insertion should reflect the transcript stability (the Insertion always combines with the 148I allele). No significant difference was observed in the Insertion carrying subjects, when compared to WT individuals: 93% (85–97%) vs. 100% (74–136%), p = 1.

## Discussion

In our study, we described and characterized a novel biallelic Indel variant in the second exon of the *PNPLA3* gene. We showed that the inserted sequence is a 5′truncated version of the LINE-1 element. LINE-1 elements are probably the only human autonomous retrotransposons that may still be active (i.e., are capable of integrating their copies at various locations throughout the genome); see Ref.^12^ for a comprehensive review. The typical LINE-1 element spans about 6 kb and comprises two open reading frames (ORF, encoding proteins responsible for reverse transcription and integration of the element) separated by a short spacer sequence and flanked with 5′ and 3′ untranslated regions (UTR)^13^. In this case, the 5′ end is largely deleted (which is quite usual^14^), resulting in an insertion just slightly over 1 kb. As most of the LINE-1 element is lost (only the 3′ UTR and a 3′ portion of ORF2 are preserved), this copy is condemned to inactivity (Fig. 2). Although the element does not disrupt the coding sequence (it integrated into the second intron of the *PNPLA3* gene, 99 bp upstream of the third exon), it may still affect gene function. Incorrect splicing/exon skipping, premature termination of transcription, or transcription initiation are some of the possible mechanisms. The impact of mobile elements on the human genome is nicely reviewed in Ref.^12^. Since only the terminal part of the LINE-1 element is present in *PNPLA3*, LINE-mediated initiation of transcription can be excluded. On the other hand, defective splicing or transcriptional disruption can not be excluded. Actually, the reverse orientation of the insert may increase the risk of introducing a polyadenylation signal and premature termination of transcription^15^. Although the number of available liver biopsies has been limited, and the data seem to be scattered (probably due to the variation of liver affections and the inhomogeneity of the tissue), our results clearly suggest no Insertion-mediated transcript modification or altered *PNPLA3* expression. The absence of any biological effect is further supported by the lack of any association between Ins allele carriage and NAFLD. Thus, the most important outcome of the Insertion seems to be the possible interference with genotyping of the clinically important I148M variant. Placing the forward primer upstream of the position of the LINE insertion would preclude amplification of the Ins-containing chromosome (the PCR product is much larger than expected), leading to selective amplification of the other allele. Due to the Ins-148I linkage, this would markedly overestimate the frequency of the causative 148M variant. In our case, the observed 148M frequency would increase from 0.3 to 0.47 (Supplementary Table 2). The most common genotyping methods in large clinical studies are TaqMan and Mass array-based assays, where primer sequences are considered proprietary and thus are not reported. Fortunately, those methods typically use very short amplicons, where the chance of incorrect placement of the forward primer is rather low (the Insert is located 122 bp upstream of the I148M variant). Other commonly used methods include PCR–RFLP or Sanger sequencing, with both prone to the Insert-mediated interference; and therefore primer placement should be carefully checked. Results of those studies using the "wrong" forward primer should be reconsidered.

In conclusion, we report the frequent LINE insertion in the second intron of the *PNPLA3* gene. Although it has no apparent biological effect, it can significantly impede the genotyping of the clinically important I148M variant in the *PNPLA3* gene. This underlines the importance of careful design and detailed reporting of genotyping methodology, including the primer sequences. Further studies are needed to assess the presence/frequency of this novel variant in non-Caucasian populations.

## Materials and methods

### Study subjects

A total of 122 NAFLD patients and 167 control subjects (all Caucasians) were included. The control subjects were unrelated healthy volunteers and employees of the First Faculty of Medicine and General Faculty Hospital. Inclusion criteria were the absence of: any apparent liver disease (indicated by physiological values of standard liver function tests), diabetes mellitus, or metabolic syndrome. NAFLD patients were recruited at the 4th Department of Internal Medicine, First Faculty of Medicine and General University Hospital in Prague. The diagnosis of NAFLD was based on clinical and laboratory parameters including: (1) evidence of hepatic steatosis either by imaging (ultrasound, CT, or FibroScan^®^ with CAP) or histology; and (2) the absence of other/secondary causes of hepatic fat accumulation: viral hepatitis, drug-induced liver disease, autoimmune liver disease, biliary diseases, and inherited metabolic diseases. Alcohol abuse was excluded by the patient´s history, short questionnaire, stable gamma glutamyl transferase activities, and use of either serum carbohydrate-deficient transferrin and/or urinary ethyl-glucuronide levels, if required. The presence of any liver-related malignancy was also an exclusionary criterion^16^. Samples of liver tissue for transcript analyses were leftovers from liver biopsies that had been performed for diagnostic/research purposes during our past/ongoing studies^17–19^. A total of 19 liver biopsies were available (11 × cirrhosis, 4 × nonalcoholic steatohepatitis, 2 × hepatitis C, 2 × autoimmune hepatitis). The study was conducted according to the guidelines of the Declaration of Helsinki, and approved by the Institutional Ethics Committees: Ethics Committee of the Regional Hospital in Liberec (NUT-3/NAS, 27/3/2019); Ethics Committee of the General University Hospital, Prague (107/11, 2011; 126/14, 21/8/2014; 40/19, 18/6/2019). All participants gave informed consent.

### Sequences and nomenclature

Accession numbers of the reference sequences used are NG_008631.1 (*PNPLA3*), and AF148856.1 (LINE-1 element). The sequence of the novel variation has been submitted to GenBank and can be accessed under MW478601. Its official name according to the HGVS^20^ would thus be NG_008631.1:g.4921_4922ins[MW478601:782_1895]. For simplicity, however, we will refer to it as the "Insertion" or as the "Ins" allele throughout this manuscript. The common *PNPLA3* substitution rs738409 (NG_008631.1:g.10109C > G, NM_025225.3:c.444C > G, NP_079501.2:p.I148M) is further referenced as I148M.

### Polymerase chain reaction

PCR amplification of products up to 700 bp was performed using AptamerTaq polymerase (TopBio, Czech Republic). However, for longer amplifications either Phusion (Thermo Fisher Scientific, USA) or ALLinRPH (HighQu GmbH, Germany) polymerases were used as suggested by the supplier. The annealing temperature was optimized individually for each primer combination. Primers were synthesized by Generi Biotech (Czech Republic; sequences are listed in Supplementary Table 1), and dNTPs were from Thermo Fisher Scientific (USA).

### Genotyping

Long PCR was performed (primers 6L and 1R) to detect the novel insertion. A PCR product of 1112 bp indicated WT, while the 2232 bp product was obtained in the case of the Ins allele (Supplementary Table 3). The I148M variant was typed by PCR–RFLP. The target region was PCR amplified with primers 3L and 2R, and cleaved with BtsCI restriction endonuclease (New England Biolabs, USA, Supplementary Table 4). The combination of allele specific PCR with RFLP was used to test the linkage of I148M with the novel Insertion. The Ins allele was amplified with primers InsL and 1R, while the 1L and 1R combination was used to amplify WT. The resulting PCR products were cleaved with BtsCI to type the I148M variant. As the InsL primer is specific for the LINE-1 element (and multiple targets are interspersed throughout the genome), its concentration in the PCR mixture was increased by 5 times that of the standard protocol. Genotypes (at both loci) of the control subjects were in Hardy–Weinberg equilibrium.

### Sanger sequencing

The PCR products were purified using the Exo/SAP method (both the Exonuclease I and Shrimp Alkaline Phosphatase were from Thermo Fisher Scientific, USA), and sequenced in both directions by SEQme (Czech Republic).

### Oxford nanopore sequencing

The target region was PCR amplified (primers 6L and 1R, ALLinRPH polymerase, 30 cycles). The resulting PCR product was checked by agarose electrophoresis, purified, and quantified on a Qubit fluorometer (Thermo Fisher Scientific, USA) according to standard procedures. The sequencing library was prepared using a 1D Ligation Sequencing kit (SQK-LSK108, Oxford Nanopore Technologies, UK). The library was run on a MinION device using the FLO-MIN107 R9 Flow Cell, according to the manufacturer´s instructions (Oxford Nanopore Technologies, UK). Base-calling was performed using ONT Albacore software v.2.1.10. The reads were assembled using Canu software v.1.7.1^21^.

### Quantitative RT-PCR

Total RNA from liver biopsies that were stored in RNAlater (Sigma-Aldrich, USA) was isolated by a GenUP Total RNA Kit (Biotechrabbit GmbH, Germany), and its integrity was assessed using agarose electrophoresis. cDNA was generated using a High-Capacity cDNA Reverse Transcription Kit (Thermo Fisher Scientific, USA), and stored at − 80 °C until analysis. Quantitative PCR was run on a ViiA 7 Real-Time PCR System using SYBR Select Master Mix chemistry (Thermo Fisher Scientific, USA). To prevent gDNA amplification, intron skipping primers were designed (for sequences see Supplementary Table 1). All samples were run in triplicates, and the melting curve was inspected. The ∆∆Ct relative quantification procedure was used throughout. Depending on the application, either the *HPRT*, 5′ region or WT allele of the *PNPLA3* transcript were used as references. Relative qPCR results are expressed as the median (IQR).

### Statistical analyses

Haplotype analysis and case–control comparisons were done using the SHEsis platform (http://analysis.bio-x.cn/myAnalysis.php)^22,23^. Reported p-values (both in single site and haplotype association analyses) were calculated by the Fisher exact test. Differences in *PNPLA3* expression were assessed by the Mann–Whitney U test (Statistica software, version 14, StatSoft, USA). p-values < 0.05 were considered significant.

## Supplementary Information

Supplementary Information.
