# Supplementary material for: Clinically silent LINE 1 insertion in the PNPLA3 gene may impede genotyping of the p.I148M variant
Source: Sci Rep. 2021 Oct 22;11:20924. doi: 10.1038/s41598-021-00425-0 (PMC8536765; doi:10.1038/s41598-021-00425-0)
Supplement: Supplementary file 1 — Supplementary Information. [file 41598_2021_425_MOESM1_ESM.pdf]

Clinically silent LINE 1 insertion in the *PNPLA3* gene may impede genotyping of the p.I148M variant.

Martin Leníček[1, *], Václav Šmíd[2], Petr Pajer[3], Anna Nazarova[1], Karel Dvořák[2,4], Iva Subhanová[1], Radan Brůha[2], Libor Vítek[1, 2]

Supplemental Table 1

Primer sequences

Name	Sequence 5´-3´
1L	ttggtcctgtctgtggtgac
1R	ccagctgtggctactctgtc
2L	ttggtcctgtctgtggtgat
2R	aaggagggataaggccactg
3L	cctgctcacttggagaaagc
3R	cgcctggccaacatcatatag
4L	gcttattggaatggcagacc
4R	aaatggagtccttcctccaag
5L	cccctgtgccagagtgac
5R	atccatgggtcaaaagaacg
6L	acctgtcctgcaggtgtgc
6R	caaacttgaaaatgcccttg
7L	cctgtatcctcagggtctagc
Ins L	ttttgttgccattgcttttg
Ex1L	gcgtcctctccggtatcc
Ex2-3L-I	cttggtatgttcctgcttcatc
Ex2-3L-M	cttggtatgttcctgcttcatg
Ex2L	agtgtctgatggggaaaacg
Ex2R	gaaggatggatggaagatgc
Ex4R	acacggtgatggttgttttg
Ex7L	tgtaccctgcctgtggaatc
Ex8R	tcgagtgaacacctgtgagg
HPRT L	cactggcaaaacaatgcagac
HPRT R	gggtccttttcaccagcaag

Supplemental Table 2

Overview of I148M and Ins/WT genotypes in all subjects

	Ins/Ins	Ins/WT	WT/WT
148 I/I	42*	77	25
148 I/M	0	71*	46
148 M/M	0	0	28*

Asterisks indicate groups where genotyping of the I148M variant would be affected by wrong forward primer placement (see Discussion). PCR amplification in compound homozygotes (Ins/Ins-148I/I) would completely fail, while all compound heterozygotes (Ins/WT-148I/M) would be incorrectly identified as 148M homozygotes. As a result, the observed frequency of the 148M variant would increase from 0.3 to 0.47.

Supplemental Table 3

PCR genotyping of the novel insertion

		1x	7.5x
Phusion GC buffer	5x	4	30
dNTPs	2mM	2	15
Primer 6L	10uM	0.4	3
Primer 1R	10uM	0.4	3
Water		12	90
Phusion polymerase	1U/ul	0.2	1.5
Master Mix		19	142.5
Template		1	7.5
Total		20	150

98°C	1 min	
98°C	20 s	25x
64°C	20 s	
72°C	2 min 45 s	
72°C	5 min	

The expected PCR product size is 1112 bp and 2232 bp for wild-type and Ins allele, respectively.

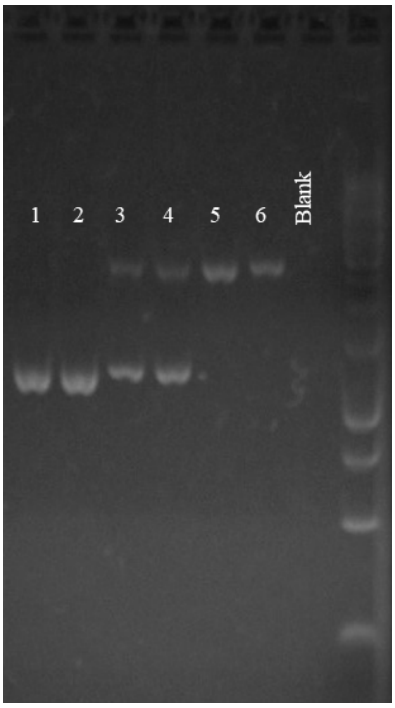

Two individuals are wild type homozygotes (lines1, 2), two are heterozygotes (lines 3, 4), and two are homozygous carriers of the novel insertion (lines 5, 6).

Supplemental Table 4

PCR-RFLP genotyping of the p.I148M variant

PCR

		1x	7.5x
Buffer wo Mg	10x	2	15
dNTPs	2mM	2	15
$MgCl_2$	25mM	2	15
Primer 3L	10uM	0.4	3
Primer 2R	10uM	0.4	3
Water		11.2	84
Aptamer-Taq polymerase	1U/ul	1	7.5
Master Mix		19	142.5
Template		1	7.5
Total		20	150

95°C	3 min	
95°C	30 s	35x
60°C	30 s	
72°C	10 s	
72°C	5 min	

The expected PCR product size is 139bp.

Restriction

		1x	7x
Cut Smart buffer	10x	1.2	8.4
Water		9.65	67.55
BtsCI	20U/ul	0.15	1.05
Master Mix		11	77
Template		4	28
Total		15	105

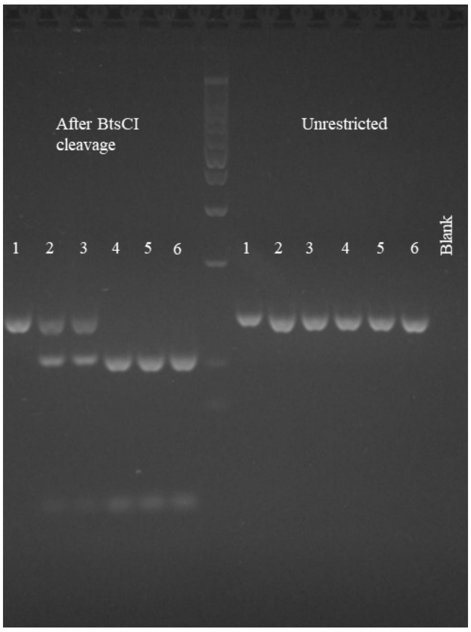

Incubation 1 hr at 50°C.

While the 148M allele is not cleaved, wild-type 148I is cut into two fragments of 107 and 32 bp.

PCR-RFLP analysis identified one individual homozygous for the pathogenic variant 148M (line 1), two heterozygotes (lines2,3) and three wild type homozygotes (lines 4-6).
